# A Highly Effective, UV-Curable, Intumescent, Flame-Retardant Coating Containing Phosphorus, Nitrogen, and Sulfur, Based on Thiol-Ene Click Reaction

**DOI:** 10.3390/ma15093358

**Published:** 2022-05-07

**Authors:** Wenqian Li, Yanli Dou, Xuefei Li, Shengbo Fang, Jian Li, Quanming Li

**Affiliations:** 1 The Ministry of Education Key Laboratory of Automotive Material, College of Materials Science and Engineering, Jilin University, Changchun 130025, China; liwq1819@mails.jlu.edu.cn (W.L.); douyl@jlu.edu.cn (Y.D.); lt13252807109@163.com (S.F.); lija1619@mails.jlu.edu.cn (J.L.); 2 China Nuclear Power Technology Research Institute Co., Ltd., Shenzhen 518031, China

**Keywords:** UV-curable coating, thiol-ene click reaction, flame retardancy, natural fiber

## Abstract

In this paper, a flame-retardant, UV-cured coating was prepared on the fiber composites’ (FC) surface via a thiol-ene click reaction using pentaerythritol tetra(3-mercaptopropionate) (PETMP), triallyl cyanurate (TAC), and 2-hydroxyethyl methacrylate phosphate (PM-2). The synergistic effectiveness of phosphorus (P), nitrogen (N), and sulfur (S) was studied in detail by changing the proportion of these reactants. Sample S_4_(N_3_P_2_)_6,_ with a molar ratio of N and P elements of 3:2, and the thiol and vinyl groups of 4:6 had the highest LOI value (28.6%) and was self-extinguishing in the horizontal combustion test. It had the lowest peak heat release rate (PHRR) value (279.25 kW/m^2^) and total smoke production (2.18 m^2^). Moreover, the thermogravimetric analysis (TG) showed that the decomposition process of the coated composites was delayed. The conversion rate of the double bond and the thiol of S_4_(N_3_P_2_)_6_ was 100% and 92.0%, respectively, which showed that the cross-linked network structure was successfully formed. The tensile strength and the flexural strength of coated composites improved, and the transparency of the coating can reach 90%. These characteristics showed that the UV-cured coatings could be used in industrial production to effectively prevent fires.

## 1. Introduction

A UV-curable coating can be used on different substrates [1,2,3,4] to enhance the materials with various properties, such as fire resistance [5,6], anti-fouling [7], and good optical transparency [8]. Compared with ordinary polymer coatings, UV-curable coatings have the advantages of short reaction time, low energy consumption, and simple operation [9]. The functional UV-curable coating is based on the modification of unsaturated resin—unsaturated polyester, acrylic resin, etc. These systems have problems that need to be solved urgently, such as high viscosity, large volume shrinkage after curing, oxygen inhibition, and a non-uniform crosslinking network [10,11]. Among them, oxygen inhibition is the main obstacle in the UV-cured process [12,13]. Oxygen reacts with the free radicals initiated by the photoinitiator in the chain growth step and finally generates peroxy free radicals with lower activity, which leads to the failure of the curing of the coating surface and the low conversion rate of the UV-cured film. So far, the most commonly used method in the industry is curing under an N_2_ atmosphere, using more photoinitiators or using multiple initiators to form a photoinitiating system [12,14]. However, these methods lead to a sharp increase in curing costs and greater volume shrinkage of the coating, which results in a decrease of the adhesion of UV-cured coatings, an increase of internal stress, and the deterioration of mechanical properties [11].

The thiol-ene click reaction uses vinyl monomers and thiol monomers as prepolymers. The polymerization reaction is a free-radical step-growth reaction with the participation of photoinitiators. The polymer molecular weight gradually increases, which greatly improves the double bond and thiol conversion rate. In this curing process, the thiol group and the peroxy radical undergo a hydrogen extraction reaction to produce a highly reactive thiol group, which effectively eliminates the problem of oxygen inhibition [1,11,15]. Moreover, the gradual growth mechanism of the thiol-ene click reaction leads to a delay in the gel point of the cured product, which can effectively release the shrinkage stress. Besides, the thiol-ene click reaction has the advantages of fast reaction speed and high selectivity. It has a non-negligible application prospect in the field of UV-cured coatings [16,17], and it is considered to be an effective method for preparing cross-linked network coatings [18,19].

To develop a green halogen-free flame-retardant coating on the surface of the natural fiber composites, the prepolymers containing N, P, and S are cross-linked via the thiol-ene click reaction under UV irradiation in this paper. Pentaerythritol Tetra(3-mercaptopropionate) (PETMP), a kind of tetramercaptan monomer, was used as a film-forming agent, due to its good reactivity with the double bonds. Meanwhile, the S element in PETMP and the N element in triallyl cyanurate (TAC) could be used as the blowing agent to build an intumescent flame-retardant system, together with the phosphorous-containing monomer 2-hydroxyethyl methacrylate phosphate (PM-2). The effect of the relative ratio between N, P, and S elements on the flame-retardant effect of the coating was studied more precisely. At the same time, the effect of the relative ratio of double bonds and sulfhydryl groups on the conversion rate of the coating and the enhancement of the mechanical properties of the composite by the presence of the coating was also investigated.

In this paper, this coating was applied on the natural fiber composites, which not only improved the flame-retardant properties of the composites but also had good adhesion and did not have a negative impact on the mechanical properties. The curing reaction kinetics and the functional group changes of the coating before and after curing were evaluated by Fourier transform infrared spectroscopy (FTIR). The flame retardancy of the coated composites was investigated by limit oxygen index (LOI), horizon burning rate (HBR), and the cone calorimetry tests (CCT), respectively. The thermal properties of the coated composites were studied by thermogravimetric analysis (TGA). The mechanical properties of the coated composites were characterized by the tensile strength, the flexural strength, and the adhesion test. Finally, the scanning electron microscope/energy-dispersive spectrometer (SEM/EDS) was used to observe the surface morphology of the char layer structure of the samples after burning and acquired the content of C, O, N, P, and S of the char layers. To sum up, this work offered an efficient, quick, convenient, and simple method for preparing crosslinked flame-retardant coatings.

## 2. Experiment

### 2.1. Materials

**Figure 1:**
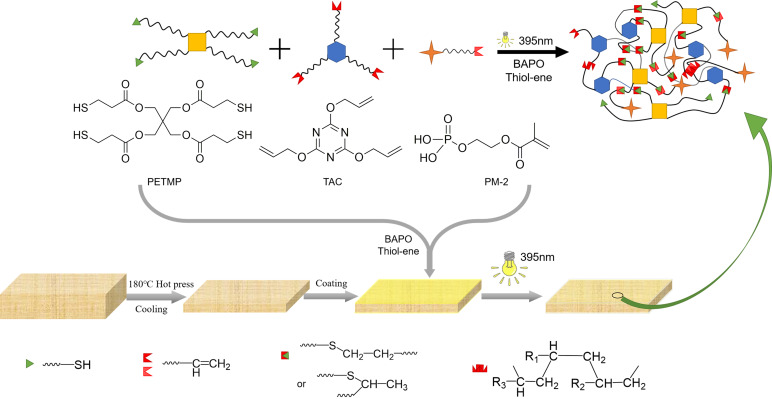
The chemical structures of Pentaerythritol tetra(3-mercaptopropionate) (PETMP), triallyl cyanurate (TAC), and 2-hydroxyethyl methacrylate phosphate (PM-2), and a brief description of the preparation for the ideal cross-linked network.

Pentaerythritol tetra(3-mercaptopropionate) (PETMP, 98.0%) was purchased from Shanghai Yuhong Chemical Technology Co., Ltd. (Shanghai, China); triallyl cyanurate (TAC, 98%) was obtained from Shanghai Aladdin biochemical technology Co., Ltd. (Shanghai, China); 2-hydroxyethyl methacrylate phosphate (PM-2, 99%) was received from Guangzhou Lihou Trading Co., Ltd. (Guangzhou, China); and phenylbis(2,4,6-trimethylbenzoyl)phosphine oxide (BAPO, 98%) was supplied by Changshu Hengyao New Material Co., Ltd. (Suzhou, China). The UV absorption peaks of the photoinitiator BAPO are 250 nm and 370 nm. All reagents were analytical grade, and they were used as received without further purification. The chemical structures of raw materials are shown in Figure 1.

### 2.2. Preparation of Samples

The flax fiber, kenaf fiber, and polypropylene fiber were blended in the mass ratio of 1:1:2 to prepare the needle-punched nonwoven felts. The non-woven felts were hot-pressed into fiber composites (FC) at 180 °C and 8 MPa. The formulation of UV curable coatings is shown in Table 1. The coatings were obtained by mixing four materials in different proportions, which included a prepolymer containing thiol groups (PETMP), a prepolymer TAC containing nitrogen, a phosphate functional monomer (PM-2), and the photoinitiator BAPO. There is one C=C bond in PM-2 and three C=C bonds in TAC; the thiol monomer contains four thiol groups, and the ratio of monomers meets C=C: SH=1:1. From this, the best ratio of nitrogen and phosphorus can be explored. On this basis, the relationship between the ratio and the conversion rate can also be studied by changing the ratio of C=C and SH. Thereafter, the prepolymer solution was mixed thoroughly with the photoinitiator BAPO (3 wt% of the total weight of TAC, PM-2, and PETMP) at 60 °C for 30 min to obtain a clear liquid. The homogeneous mixture was coated on the fiber composites with a 200 μm film-applicator to form uniform films. Samples were cured under a 395 nm light from a UV lamp (Zhongshan Guzhen Yanxizhao Lighting Appliance Factory, AC90V-240V 395 nm 35W, Zhongshan, China) at a distance of 10 cm for 1 min or more to ensure an adequate click reaction. The structure of the prepolymers and a brief description of preparation for an idealized cross-linked network via a thiol-ene click reaction is shown in Figure 1.

### 2.3. Characterization

#### 2.3.1. Fourier Transform Infrared Spectroscopy (FTIR)

The photopolymerization kinetics were studied by the TENSOR 27 FTIR spectrometer (Germany Bruker, Berlin) in the range of 4000–400 cm^−1^. The double bond conversation was monitored by measuring the area under the peak of 1637 cm^−1^, and the peak at 2572 cm^−1^ was used to measure the thiol group conversation. The peak at 1730 cm^−1^ represented the stretching vibration peak of C=O, and the intensity of the peak did not change significantly during the FTIR tests. As a result, the peak at 1730 cm^−1^ was used as an internal standard peak to neutralize the variation in the coatings caused by rapid curing [2,5,20]. The double bond conversation rate (DC) could be calculated according to Equation (1):

(1) $$\mathrm{DC}\;(\%)=\left(1\;-\;\frac{A_{t}\left(1637{\mathrm{cm}}^{-1}\right)/A_{t}\left(1730{\mathrm{cm}}^{-1}\right)}{A_{0}\left(1637{\mathrm{cm}}^{-1}\right)/A_{0}\left(1730{\mathrm{cm}}^{-1}\right)}\right)\;\times \;100\%$$

The thiol group conversation (TC) could be calculated according to Equation (2):

(2) $$\mathrm{TC}\;(\%)=\left(1\;-\;\frac{A_{t}\left(2572{\mathrm{cm}}^{-1}\right)/A_{t}\left(1730{\mathrm{cm}}^{-1}\right)}{A_{0}\left(2572{\mathrm{cm}}^{-1}\right)/A_{0}\left(1730{\mathrm{cm}}^{-1}\right)}\right)\;\times \;100\%$$

where A_0_(1637 cm^−1^), A_0_(1730 cm^−1^), and A_0_(2572 cm^−1^) represent the integral peak area of C=C, C=O, and -SH, before curing, respectively; the t at A_t_ represents the UV-curing time.

#### 2.3.2. Limiting Oxygen Index (LOI)

The LOI of the coated composites was obtained by an LOI instrument (JF-3, Seoul, Korea FESTEC Company) according to GB/T 2406.2-2009 (China) oxygen index method. The sample size used for the test was 100 × 10 × 3 mm^3^.

#### 2.3.3. Horizontal Burning Rate (HBR)

The HBR test was performed using a burning test instrument (H1011D, ChangchunshiHosly apply technique graduate school, Changchun, China). The test was measured according to the Flammability of automotive interior materials (GB 8410-2006). The composites were ignited for 15 s, and then the fire was removed. Burning length and time were recorded to calculate the burning rate. The sample size used for HBR tests was 150 × 75 × 3 mm^3^.

#### 2.3.4. Thermo-Gravimetric Analysis (TG)

The thermogravimetric (TG) analysis results were obtained on a Q500 thermal analyzer (TA Instruments, New Castle, DE, USA) under a nitrogen atmosphere (50 mL/min), and under an air atmosphere with a heating rate of 10 °C/min. About 5–10 mg of the samples was placed in an alumina crucible and heated from 25–800 °C.

#### 2.3.5. Cone Calorimetry Test (CCT)

The CCT is one of the best methods to evaluate the combustion characteristics of the samples. The CCT was performed with a cone calorimeter apparatus (FTT0242, West Sussex, UK) according to ISO 5660 (2002). In the test, the coated composites were exposed to a radiation cone with a heat flux of 50.0 kW/m^2^. The sample size was 100 × 100 × 3 mm^3^.

#### 2.3.6. Mechanical Properties

The mechanical tests, including the tensile strength and the bending strength, were performed using a universal testing machine WSM-5KN (Changchun intelligent instrument equipment Co., Ltd., Changchun, China) according to the TL52448. The dimensions of each specimen were 100 × 25 × 3 mm^3,^ and the strain rate was 3 mm/min. All mechanical data had an effective average of 5.

#### 2.3.7. Adhesion Test

The adhesion test was measured using the single blade pocket knife in accordance with ISO 2409:2013. The number of cuts in each direction was 6, and the cross-cut spacing was 3 mm.

#### 2.3.8. Optical Transparency

The optical transparency of the UV-cured coatings was recorded using a UV-6100s Scanning UV-Vis Spectrophotometer (Shanghai Mepda Instrument Co., Ltd., Shanghai, China) in the range of 200–1100 nm.

#### 2.3.9. Morphology Observation

The char residues after HBR tests were observed via scanning electron microscopy (SEM, 30 kv/Magellan400, FEI NanoPorts, Hillsboro, Oregon, USA). The energy-dispersive spectrometer (EDS, Magellan400, FEI NanoPorts, Hillsboro, Oregon, USA) on the SEM was used to observe the surface of the coated samples after HBR tests and compare the content of C, O, N, P, and S of the coatings on the surface of coated composites before and after the HBR test.

## 3. Results and Discussion

### 3.1. Curing Kinetics and Double Bond Conversion

**Figure 2:**
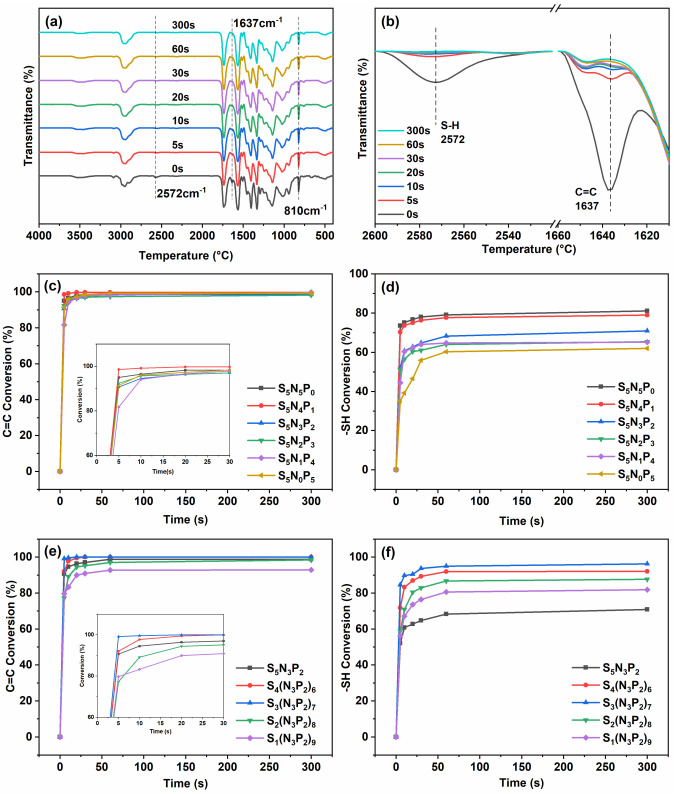
The Fourier Transform Infrared Spectroscopy (FTIR) spectra of sample S_4_(N_3_P_2_)_6_, which were recorded during the UV-curing (**a**); the enlarged FTIT spectra of the thiol group (2572 cm^−1^) and the acrylate double bond (1637 cm^−1^) of the coating S_4_(N_3_P_2_)_6,_ after being subjected to UV-irradiation for various durations (**b**); the double bond conversion rate versus the irradiation time (**c**,**e**); and the thiol group conversion versus the irradiation time (**d**,**f**).

In order to study the effect of the relative changes of ratio of -SH to C=C and the TAC to PM-2 on the curing performance of the coating, the FTIR technique was employed to monitor the chemical structure changes of the S_5_N_x_P_y_ and S_x_(N_3_P_2_)_y_ series coatings before and after curing. As shown in Figure 2, the FTIR spectra of sample S_4_(N_3_P_2_)_6_ was recorded at different UV-irradiation times (5 s, 10 s, 20 s, 30 s, 60 s, and 300 s) [2,4,20,21]. The conversion rate of the thiol and vinyl groups gradually stabilized after 20 s. Besides, the C=C and -SH peak intensity remained almost unchanged at 60–300 s, which indicated that the curing process was complete and the double bond and the thiol had formed the cross-linked network structure. Thus, the coating formed when the curing time was 60 s.

Figure 2c showed that the relative change of PM-2 and TAC content had little effect on the double bond conversion rate. However, when the -SH and C=C molar ratio was 1:1, as the content of PM-2 increased, the -SH conversion rate gradually decreased as in Figure 2d. This may be because there were acrylate groups in PM-2; the double bond tended to undergo homopolymerization and does not copolymerize with thiol during the UV-curing process, which led to a higher double bond conversion rate than the thiol conversion rate (Figure 2c,d). The final conversion rate of thiol and double bond is shown in Table 2; the conversion rate of the double bond of coating S_3_(N_3_P_2_)_7_ and S_4_(N_3_P_2_)_6_ reached 100%; and the conversion rate of thiol reached 96.2% and 92%, respectively. The double bond and thiol had an optimal ratio of homopolymerization and copolymerization, and the coatings had good film-forming properties. However, excessive C=C content led to homopolymerization and lower thiol conversion rate (Figure 2e,f), and the -SH conversion rates of samples S_1_(N_3_P_2_)_9_ and S_2_(N_3_P_2_)_8_ decreased to 87.6% and 81.8%, respectively. At this time, the oxygen inhibition effect in the homopolymerization reaction gradually appeared, so the double bond conversion rate also decreased to a certain extent. However, the double bond conversion rate of sample S_1_(N_3_P_2_)_9_ remained at 92.8%. This showed that the presence of -SH can effectively counter oxygen inhibition and ensure the coating has good film-forming properties. To sum up, the entire coatings system had good curing efficiency, good film-forming performance, and could form a complete cross-linked network structure.

### 3.2. Flame Retardancy of Fiber Composites

**Figure 3:**
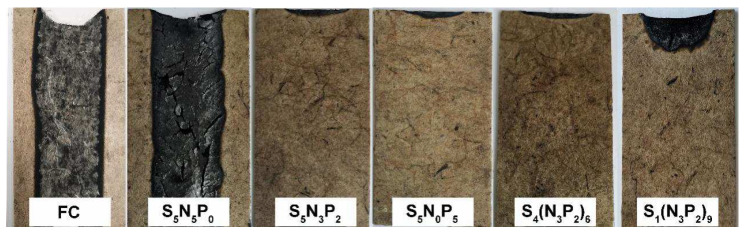
The residual carbon layer sample after a horizontal combustion test.

The formulation of the coating is the main factor affecting the flame-retardant performance. From Table 2, the proportion of flame-retardant elements was appropriate when the molar ratio of N and P was 3:2, and it played a synergistic flame-retardant role. The sample S_5_N_3_P_2_ had the best flame-retardant performance among the S_5_N_x_P_y_ series. The LOI value reached 27.2%, and was extinguished after 79 s. In addition, when the molar ratio of SH and C=C was 4:6, sample S_4_(N_3_P_2_)_6_ had the highest LOI value, up to 28.6%. In the horizontal combustion test, it quickly extinguished after being ignited for 85 s, which showed that S could also be used as a flame-retardant element and synergistically a flame-retardant with N and P elements. Figure 3 showed the carbon residue morphology of some samples after the horizontal combustion test. It can be seen that samples FC and S_5_N_5_P_0_ were not self-extinguishing during the horizontal combustion test. The FC carbon layer was an amorphous and loose coke residue, and no dense carbon layer was formed. Sample S_5_N_5_P_0_ formed a part of the carbon layer on the surface, but the carbon layer was disconnected and broken and thus did not effectively prevent the material from burning. The samples containing P elements all self-extinguished, indicating that P elements have obvious solid-phase flame-retardant effects. In general, when the N, P, and S elements coexisted in the coating, it showed an obvious synergistic effect of the gas-phase flame retardant and a solid-phase flame retardant, and it obtained better flame retardancy.

### 3.3. Combustion Behavior

**Figure 4:**
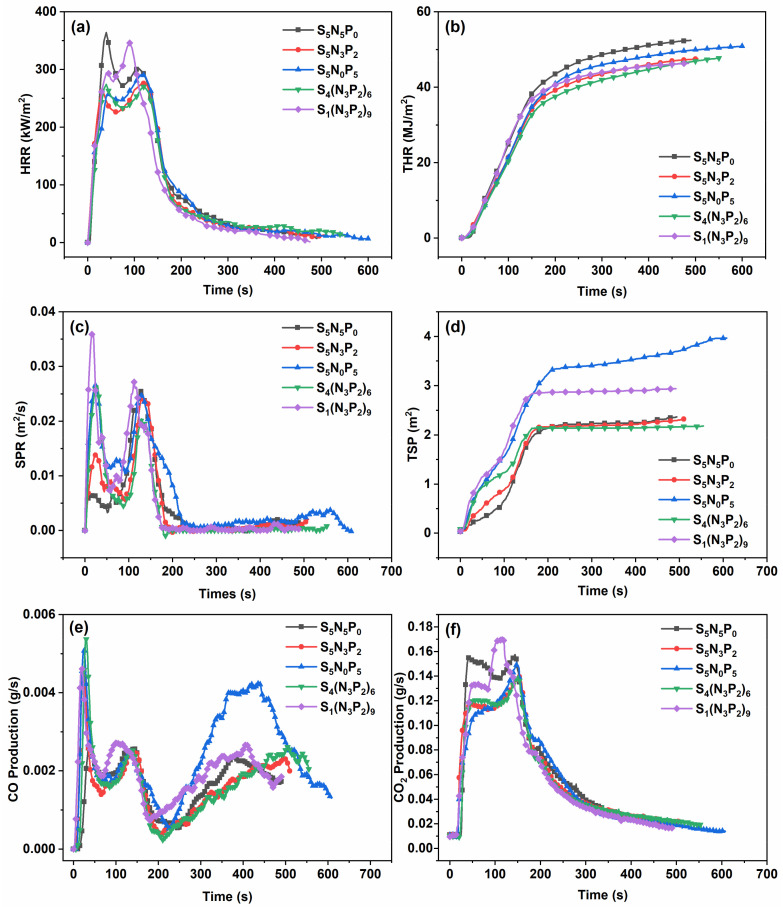
(**a**) heat release rate (HRR), (**b**) total heat release rate (THR), (**c**) the smoke production rate (SPR), (**d**) the total smoke release (TSP), (**e**) CO production, and (**f**) CO_2_ production curves as functions of the combustion time for the coated fiber composites.

The cone calorimetry test can obtain the ignition time (TTI), the heat release rate (HRR), the peak heat release rate (PHRR), the total heat release rate (THR), the effective heat of combustion (EHC), the smoke production rate (SPR), the total smoke release (TSP), the CO and CO_2_ production, and other important parameters that can evaluate the potential fire hazard of materials [4,22]. Samples S_5_N_5_P_0_, S_5_N_3_P_2_, S_5_N_0_P_5_, S_4_(N_3_P_2_)_6_, and S_1_(N_3_P_2_)_9_ were selected for cone calorimetry analysis to further study the flame-retardant properties of the coating. The test results are shown in Figure 4 and Table 3. It is worth noting that due to the presence of flame-retardant elements such as N, P, and S, the coating formed an expanded carbon layer on the surface of the composites in the combustion process. The carbon layer could be used as a good thermal insulation layer, so HRR had a downward trend in 40–70 s, resulting in the first peak of the HRR curve. Subsequently, the matrix composites gradually burned, degraded, and released flammable products from the cracks of the carbon layer under the action of heat. The existence of the fireproof layer prevented the spread of flame and the combustion of the matrix materials, which resulted in a second peak in the HRR curve. In the HRR curve, sample S_4_(N_3_P_2_)_6_ exhibited the lowest PHRR value, indicating that sample S_4_(N_3_P_2_)_6_ had a good flame-retardant effect. As shown in Figure 4b and Table 3, the THR values of all the coated composites were not much different because all samples were completely consumed during the cone calorimetry. The types of flame-retardant elements contained in the coating could slightly affect the overall flammability of the fiber composites. When the coating contained only S and N (sample S_5_N_5_P_0_) or only S and P (sample S_5_N_0_P_5_), the HRR of the composites was 52.44 MJ/m^2^ and 50.89 MJ/m^2^, which was higher than the coatings containing N, P, and S elements (around 47 MJ/m^2^). This could indicate that the three flame-retardant elements of N, P, and S had a synergistic flame-retardant effect. However, the three elements’ molar ratio had little effect on the HRR values of combustion. In addition, it must be mentioned that the TTI value of the coating samples with more phosphorus content decreased slightly, which might be attributed to the rapid rupture of the P-O-C bond and promoted the formation of the flame-retardant carbon layer.

Generally speaking, smoke and toxic gases will threaten people’s health by poisoning and suffocating them in a real-life fire scene. The curves of the SPR and TSP of the samples are shown in Figure 4c,d, and the relevant data are summarized in Table 3. The trend of the SPR curve was similar to the HRR curve. As shown in Figure 4c, the composites reached the first peak in 20 s and then decreased rapidly in 20–60 s. Sample S_4_(N_3_P_2_)_6_ had a higher initial peak value, which was mainly attributed to the violent combustion of the phosphorous-contained coatings, and then the phosphate groups formed a dense carbon layer during the combustion process. This caused the second peak of the SPR curve of sample S_4_(N_3_P_2_)_6_ to be lower than other samples. Therefore, sample S_4_(N_3_P_2_)_6_ had the lowest TSP value, reaching 2.18 m^2^. This showed that the synergistic effect of N, S, and P elements could reduce smoke production in an appropriate proportion. CO and CO_2_ production (Figure 4e,f) were also obtained to study the toxicity of burning products [22]. An increase in the CO production value of the coated composite was observed in the first 30 s, which might have been caused by rapid degradation and incomplete combustion of the coating. In the first 100 s since the formed carbon layer effectively prevented the escape of combustible gas CO, the CO production was significantly reduced within 100–200 s. However, there was a slow increase in CO production at the end of the combustion. This might have been because the early combustion consumed a lot of oxygen, resulting in insufficient oxygen content and incomplete combustion in the later stage of the combustion. Meanwhile, the TSP and CO production of the coatings with the N element were significantly reduced, indicating that the N element had an obvious gas-phase flame-retardant effect, which could effectively reduce the smoke emission. In addition, compared with other coating composites, sample S_4_(N_3_P_2_)_6_ had significantly lower CO_2_ production, which could effectively reduce the risk of suffocation in a real fire.

### 3.4. Thermogravimetric Analysis

**Figure 5:**
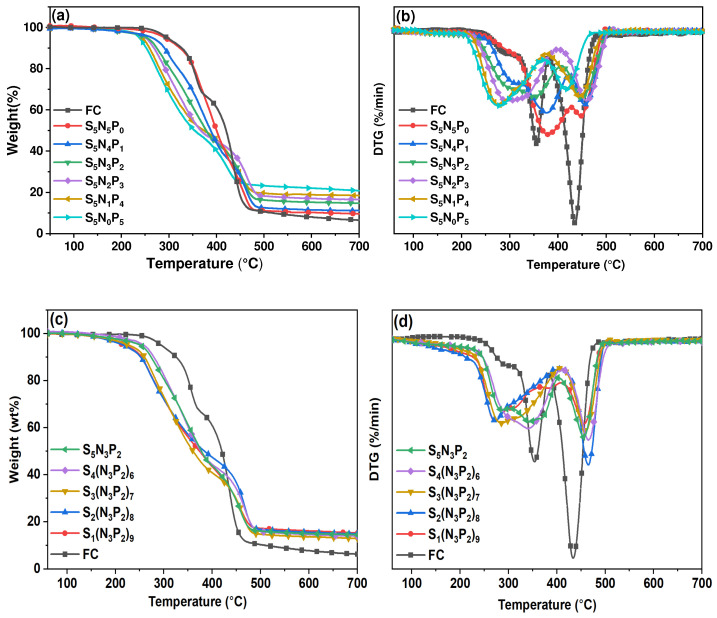
The thermogravimetric (TG) analysis (**a**) curves and differential thermogravimetric (DTG) curves (**b**) of the series of S_5_N_x_P_y_, and the TG (**c**) curves and DTG curves (**d**) of the series of S_x_(N_3_P_2_)_y_ (in an N_2_ atmosphere).

**Figure 6:**
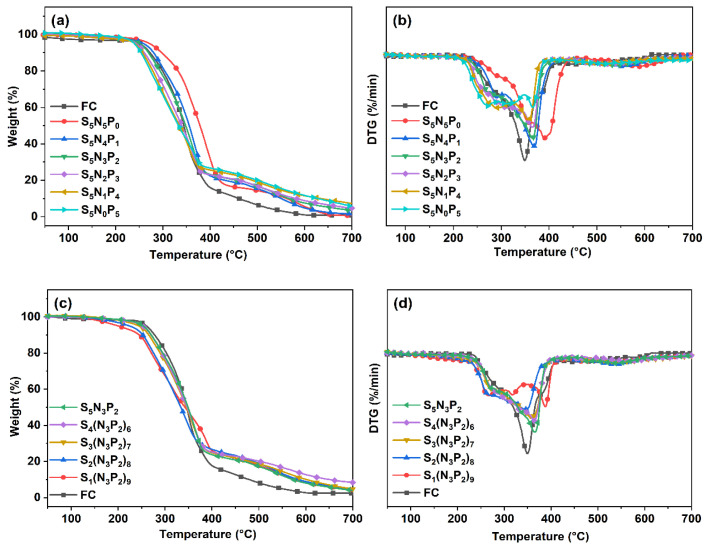
The TG (**a**) curves and DTG curves (**b**) of the series of S_5_N_x_P_y_, and the TG (**c**) curves and DTG curves (**d**) of the series of S_x_(N_3_P_2_)_y_ (in an air atmosphere).

The influence of the changes of S, N, and P elements in the coating on the thermal stability of the composite was studied by TG and DTG curves. The TG and DTG curves of UV-coated composites are shown in Figure 5 and Figure 6. Table 4 showed the thermogravimetric parameters, including the initial degradation temperature (*T*_5%_), the temperature corresponding to the maximum degradation rate (*T*_max1_, *T*_max2_), and the carbon residue Y_c_ at 700 °C. The sample FC had a high initial degradation temperature, and Y_c_ can only reach 6.55% and 0.79% in N_2_ and Air, respectively. With the increase of PM-2 content, the initial degradation temperature of the coated composites was gradually decreasing. This was mainly due to the lower bond energy of the P-O-C bond, which was not as stable as the C-C bond [23,24]. A small amount of the degradation of the coated composites below 200 °C was mainly due to the volatilization and degradation of a small part of the small-molecule impurities that were not fixed in the cross-linked network. In the DTG curve, the degradation process of FC corresponds to the thermal decomposition of hemicellulose (250–300 °C), cellulose (320–390 °C), and polypropylene (400–500 °C). The degradation peak (240–400 °C) in the first stage of the coated composites mainly corresponded to the breaking of the C-S bond in the cross-linking network, the volatilization of volatiles containing C and S (SO_2_, SO_3,_ and CO_2_), the decomposition of the phosphate group in the PM-2 structure, the scission of long chain branches in the solidified layer, and the preliminary formation of the carbon layer. The degradation of the cellulose and hemicellulose of the matrix composites was also included in this degradation peak. The degradation peak in the second stage was mainly the rupture of the cross-linked network and the main chain of the polypropylene product in the matrix material. Then, the entire matrix of the composites was almost completely degraded, leaving only part of the carbon residue [25,26]. In air atmosphere, the first degradation peak is at 200–300 °C and the second degradation peak ranges from 300–450 °C. Both decomposition stages are advanced, which indicates that the presence of oxygen accelerates the degradation process of the composite. The third degradation peak (at 500–600 °C) occurs for the thermal degradation in an air atmosphere, which is mainly attributed to the degradation of unstable carbon layers at high temperatures. It can be seen from Table 4 that, compared with S_1_(N_3_P_2_)_9_, S_4_(N_3_P_2_)_6_ and S_5_N_3_P_2_ exhibited a good synergistic flame-retardant effect due to the appropriate relative proportions of N, P, and S, and *T*_max2_ was also higher than other coating composites. The gas-phase and the solid-phase flame-retardant mechanism worked together and delayed the degradation process to a certain extent. In the S_5_N_x_P_y_ series, sample S_5_N_0_P_5_ had the highest Y_c_ at 700 °C. However, in the samples of the S_x_(N_3_P_2_)_y_ series, even if the PM-2 content increased, Y_c_ did not change significantly. This was mainly due to the decrease in the content of PETMP, which was the carbon chain provider. In addition, it can be observed from the DTG curves that due to the presence of the coating, the peak degradation rate of all coated composites was much lower than that of the uncoated fiber composite materials, which shows that the coating effectively reduced the degradation rate.

### 3.5. Char Residues Surface Morphologies Analysis and Elements Analysis

**Figure 7:**
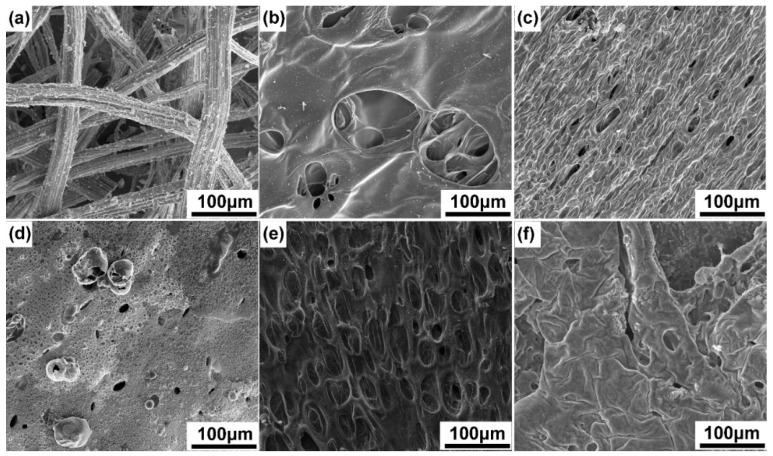
The micro-morphologies of the char layer structure of the uncoated composite FC (**a**) and the coated composites samples of S_5_N_5_P_0_ (**b**), S_5_N_3_P_2_ (**c**), S_5_N_0_P_5_ (**d**), S_4_(N_3_P_2_)_6_ (**e**), and S_1_(N_3_P_2_)_9_ (**f**), after horizontal burning rate (HBR) tests.

After the horizontal combustion test, all samples were imaged by SEM to evaluate the surface morphology and chemical composition of the residual carbon (Figure 7). According to the previous literature [27], the dense carbon layer could isolate thermal oxygen and other volatile combustible gases well, thereby improving the flame retardancy [28]. The coating with a high content of PM-2 had a complete carbon layer structure. When the content of the PETMP groups was reduced (Figure 7f), the sufficient carbon layer could not form completely on the surface of the composites. There were cracks and through holes on the carbon layer. Meanwhile, the gas-phase flame-retardant did not have a sufficient effect to achieve a relative balance with the solid-phase flame-retardant mechanism, so the flame-retardant effect gradually decreased. In contrast, in the S_5_N_3_P_2_ and S_4_(N_3_P_2_)_6_ coatings (Figure 7c,e), pores and closed bubbles appear in the carbon layer, which are caused by the release of incombustible gas during the combustion process. The carbon layer showed a honeycomb structure, which formed a temperature gradient on the carbon residue layer, inhibiting heat transfer, preventing oxygen diffusion, and providing better heat insulation performance for the base material [24,27,29,30].

**Figure 8:**
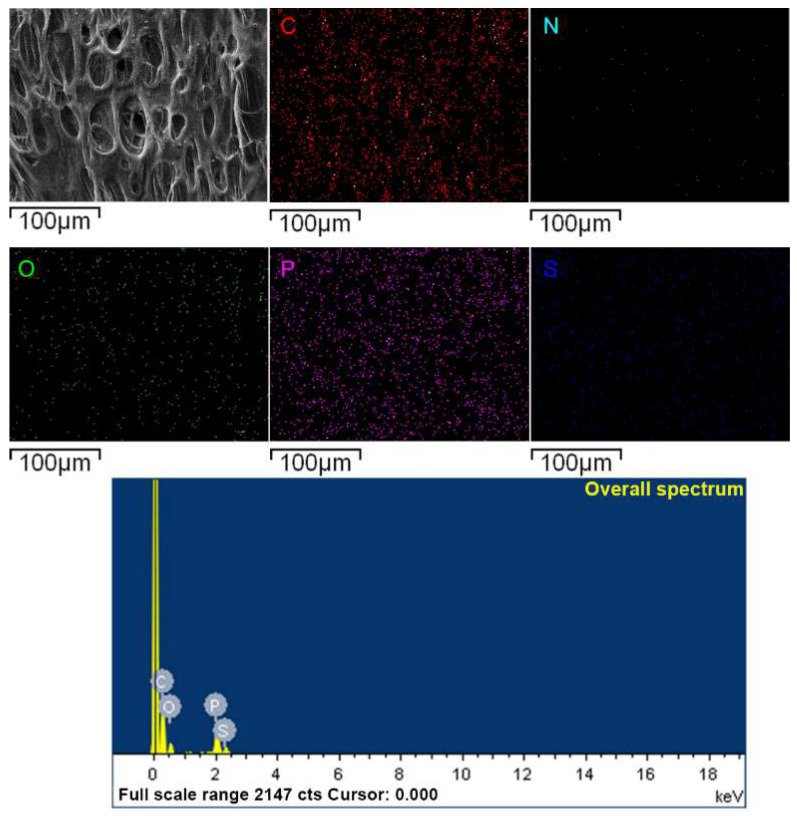
The energy-dispersive spectrometer (EDS) elements petra of sample S_4_(N_3_P_2_)_6_.

According to EDS analysis (Figure 8), the N element in samples completely disappeared, which indicated that the N element completely generated non-flammable gas during the combustion process (Table 5). The carbon residue on the surface of the S_4_(N_3_P_2_)_6_ carbon layer contained 69.53 wt% of C, 6.55 wt% of P, and 1.42 wt% of S. Compared with the element content before the combustion (Table 6), the content of S element was reduced, which showed the element S participated in the flame-retardant reaction through the gas phase flame-retardant mechanism. The phosphate group in the coatings generated polyphosphoric acid and its derivatives to promote the formation of the carbon layer during combustion. The P element in the coating was almost completely deposited in the carbon layer. In summary, the coating formed a carbonaceous layer rich in phosphorus on the surface of the composites in the combustion process, which delayed the pyrolysis reaction during the combustion process and had a good flame-retardant effect.

### 3.6. Adhesion and Mechanical Testing

Table 7 shows the tensile strength, flexural strength, and adhesion of the coated composites. When the molar ratio of -SH and C=C was 1:1, the change of TAC and PM-2 content had relatively little effect on adhesion. The coating did not fall off at all after the tests, and the adhesion grade was 0. This was mainly due to the fact that the thiol and the double bond underwent a click reaction via a free-radical step-by-step copolymerization [5,7,31,32]. The cross-linked network structure formed in this way had low shrinkage and high monomer conversion. In addition, PM-2 formed the anchor effect with PP on the surface of the composites through the penetration of its phosphate ester, so the coatings had better adhesion. Because of the formation of flexible thioether bonds, the lack of rigid structure led to the poor mechanical properties of the coating [33], so the tensile strength of the S_5_N_x_P_y_ samples did not obviously increase. However, due to the better toughness of the coating produced by this method, the bending strength fluctuated about 42.6 Mpa, which was an increase of 24.3% compared with the uncoated composites.

By fixing the relative content of TAC and PM-2 and changing the ratio of thiol to a double bond, the following dynamic emerged: the lower the thiol content, the worse the adhesion of the coating. The peeling rate of sample S_1_(N_3_P_2_)_9_ after the adhesion test even reached 48%. As the content of double bonds increases, the cross-linking of the coating tends to homopolymerize between the double bonds. The internal stress was not released due to the rapid curing of the coating, and the formed coating had a large volume shrinkage rate and high surface tension [7,34], thereby reducing the adhesion between the coating and the substrate. Additionally, the influence of the coating on the mechanical properties was related to the crosslinking method of the coating. The cross-linking density of the coating formed by the double-bond homopolymerization reaction became larger, which increased the hardness of the coating. Therefore, the tensile strength and flexural strength increased with the increase of the double bond content.

### 3.7. Optical Property

**Figure 9:**
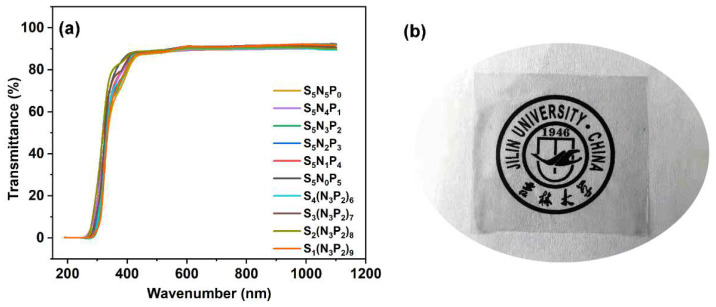
The UV-vis transmittance spectra of the UV-cured coatings (**a**), and a digital photo of sample S_4_(N_3_P_2_)_6_ (**b**).

Figure 9 is the optical transparency spectrum of the UV-cured coatings. The thickness of the coatings was 200 μm. It was obvious from the digital photos of Figure 9b that the crosslinked coating had good transparency. The UV-vis spectrum showed that the transparency of these coatings at visible light wavelengths (400–800 nm) was about 90%, which was consistent with the performance of digital photos. The transmittance of the coating was close to 0 under 300 nm UV light, which indicated that the coating had a good blocking effect on short-wave UV light [20]. The relative content changes of PETMP, PM-2, and TAC in the film had little effect on the optical transparency of the UV curable film [5]. This kind of film with high transparency showed obvious application potential.

## 4. Conclusions

The UV-cured, flame-retardant coatings containing N/P/S were successfully prepared on the surface of the composites via a thiol-ene click reaction. The effect of the element ratio on the flame-retardant properties of the coating was investigated. Among the PETMP/TAC/PM-2 crosslinked networks, sample S_4_(N_3_P_2_)_6_ showed the best flame retardancy, which was determined by LOI, HBR, and CCT. The LOI value of sample S_4_(N_3_P_2_)_6_ reached 28.6%, and its self-extinguishing time was 85 s in the horizontal combustion test. Meanwhile, it had the lowest THR value, the lowest PHRR value, and the lowest TSP value. All these results confirmed the synergistic effectiveness of flame retardants between PETMP, TAC, and the PM-2. The thermal degradation results of the UV-cured coatings revealed that the P-containing prepolymers could promote the carbon chain into a compact and stable char layer in a timely fashion, which demonstrated that P and C exert a synergistic flame retardancy in the condensed phase. Moreover, the coatings consumed oxygen and generated CO_2_ and SO_2_, and N_2_ and NH_3_, which could dilute combustible gas produced during combustion around the fiber composites. This indicated that N and S exert flame retardancy in the gas phase, leading to the formation of the honeycomb porous carbon layer. This carbon layer was monitored in the SEM test. In addition, the UV-curable coating S_4_(N_3_P_2_)_6_ also had a high double bond conversion rate and thiol conversion rate (100% and 92%, respectively) and had good mechanical properties. The flexural strength of the composites was increased to 42.02MPa. The coating had good adhesion, and the shedding rate was within 5%. The transparency of this coating was about 90%, and it had good optical properties. The aforementioned results indicated that the PETMP/TAC/PM-2 cross-linked network had potential in the flame-retardant coating field. Therefore, this research provided new ideas for the development of flame-retardant coatings that meet the needs of practical applications.

## Figures and Tables

**Table 1:** The formulation of the Ultraviolet (UV)-curing films.

Coating	PETMP (mol)	TAC (mol)	PM-2 (mol)	[SH]: [C=C] (mol)	N:P (mol)
S_5_N_5_P_0_	3	4	0	5:5	5:0
S_5_N_4_P_1_	3	3.2	2.4	5:5	4:1
S_5_N_3_P_2_	3	2.4	4.8	5:5	3:2
S_5_N_2_P_3_	3	1.6	7.2	5:5	2:3
S_5_N_1_P_4_	3	0.8	9.6	5:5	1:4
S_5_N_0_P_5_	3	0	12	5:5	0:5
S_4_(N_3_P_2_)_6_	1.5	1.8	3.6	4:6	3:2
S_3_(N_3_P_2_)_7_	1.5	2.8	5.6	3:7	3:2
S_2_(N_3_P_2_)_8_	1.5	4.8	9.6	2:8	3:2
S_1_(N_3_P_2_)_9_	1.5	10.8	21.6	1:9	3:2

**Table 2:** The properties of the UV-cured, flame-retardant coatings.

Coating	LOI (%)	HBR (mm/min) or Self-Extinguishing Time (s)	C=C Conversion (%)	-SH Conversion (%)
FC	22.8	14.3 mm/min	-	-
S_5_N_5_P_0_	25.6	5.8 mm/min	98.6	81.1
S_5_N_4_P_1_	26.7	125 s	99.7	79.0
S_5_N_3_P_2_	27.2	79 s	98.8	70.9
S_5_N_2_P_3_	26.9	94 s	98.2	65.4
S_5_N_1_P_4_	26.5	94 s	99.7	65.2
S_5_N_0_P_5_	26.3	91 s	99.3	62.0
S_4_(N_3_P_2_)_6_	28.6	85 s	100	92.0
S_3_(N_3_P_2_)_7_	27.0	140 s	100	96.2
S_2_(N_3_P_2_)_8_	25.9	214 s	97.1	87.6
S_1_(N_3_P_2_)_9_	25.5	220 s	92.8	81.8

**Table 3:** The cone calorimetry data of the coated composites.

Samples	TTI	PHRR	Mean EHC	THR	TSR	TSP
S_5_N_5_P_0_	20	384.42	21.33	52.44	243.55	2.36
S_5_N_3_P_2_	11	294.55	21.37	47.57	254.18	2.32
S_5_N_0_P_5_	10	296.37	19.84	50.89	434.56	3.96
S_4_(N_3_P_2_)_6_	18	279.25	23.73	47.80	217.03	2.18
S_1_(N_3_P_2_)_9_	7	362.53	20.26	46.41	321.83	2.93

**Table 4:** The thermal properties of the UV-cured composites.

Samples	*T*_5%_ (°C)		*T*_max1_ (°C)		*T*_max2_ (°C)		Y_c_ (wt%)	
	N_2_	Air	N_2_	Air	N_2_	Air	N_2_	Air
FC	296	251	355	-	435	349	6.55	0.79
S_5_N_5_P_0_	291	271	378	285	450	390	9.59	0.69
S_5_N_4_P_1_	265	259	376	291	458	368	11.14	1.51
S_5_N_3_P_2_	252	251	350	271	456	366	14.49	3.50
S_5_N_2_P_3_	251	243	291	290	464	365	16.53	4.64
S_5_N_1_P_4_	244	240	277	291	449	357	18.41	6.68
S_5_N_0_P_5_	237	239	276	269	422	366	20.78	5.82
S_4_(N_3_P_2_)_6_	258	252	342	280	465	365	13.76	8.46
S_3_(N_3_P_2_)_7_	238	244	285	271	459	357	14.10	4.88
S_2_(N_3_P_2_)_8_	219	224	272	264	463	345	14.94	4.63
S_1_(N_3_P_2_)_9_	225	200	271	264	456	388	14.49	4.37

**Table 5:** The element percentage content of the char layer surface of S_5_N_0_P_5_, S_5_N_3_P_2_, S_5_N_5_P_0_, S_4_(N_3_P_2_)_6,_ and S_1_(N_3_P_2_)_9,_ according to EDS tests.

Coating	C	O	N	P	S
S_5_N_5_P_0_	83.73	11.89	0	0.5	3.88
S_5_N_3_P_2_	77.37	14.91	0	4.97	2.75
S_5_N_0_P_5_	59.63	26.25	0	12.72	1.41
S_4_(N_3_P_2_)_6_	69.53	22.51	0	6.55	1.42
S_1_(N_3_P_2_)_9_	68.79	20.71	0	10.5	0

**Table 6:** The mass fractions of N, P, and S contained in films of S_5_N_0_P_5_, S_5_N_3_P_2_, S_5_N_5_P_0_, S_4_(N_3_P_2_)_6,_ and S_1_(N_3_P_2_)_9_.

Coating	C-Content (wt%)	O-Content (wt%)	N-Content (wt%)	P-Content (wt%)	S-Content (wt%)
S_5_N_5_P_0_	48.23	23.39	6.82	0	15.59
S_5_N_3_P_2_	42.41	31.27	3.28	4.85	12.51
S_5_N_0_P_5_	37.05	38.55	0	9.34	9.64
S_4_(N_3_P_2_)_6_	42.58	32.23	3.90	5.76	9.92
S_1_(N_3_P_2_)_9_	42.93	34.99	5.70	8.42	2.41

**Table 7:** The mechanical properties of the coated composites and the adhesion grade of the coatings.

Samples	Tensile Strength (MPa)	Flexural Strength (MPa)	Adhesion (Grade)	
			Coating Loss Rate (%)	Adhesion Grade
FC	26.05 ± 0.85	34.27 ± 2.50	-	-
S_5_N_5_P_0_	24.85 ± 2.55	44.65 ± 1.45	0	0
S_5_N_4_P_1_	25.87 ± 3.57	44.39 ± 2.62	0	0
S_5_N_3_P_2_	25.76 ± 3.82	42.52 ± 2.56	0	0
S_5_N_2_P_3_	24.38 ± 2.60	39.96 ± 3.35	0	0
S_5_N_1_P_4_	25.24 ± 2.54	45.15 ± 2.81	0	0
S_5_N_0_P_5_	24.15 ± 2.61	40.90 ± 2.36	0	0
S_4_(N_3_P_2_)_6_	26.11 ± 1.83	42.02 ± 2.74	3	1
S_3_(N_3_P_2_)_7_	28.34 ± 2.52	48.11 ± 1.64	5	1
S_2_(N_3_P_2_)_8_	28.95 ± 0.53	50.29 ± 2.29	14	2
S_1_(N_3_P_2_)_9_	30.67 ± 2.63	52.31 ± 1.98	48	4

## Data Availability

The data presented in this study are available upon request from the corresponding author.
